# Comparison of colorimetric loop-mediated isothermal amplification kit and reverse transcription-polymerase chain reaction in the diagnosis of peste des petits ruminants in sheep and goats in Southeast Nigeria

**DOI:** 10.14202/vetworld.2020.2358-2363

**Published:** 2020-11-07

**Authors:** Ijeoma Chekwube Chukwudi, Kenneth Ikejiofor Ogbu, Pam Dachung Luka, Refiloe Petunia Malesa, Livio Edward Heath, Emmanuel Ikenna Ugochukwu, Kennedy Foinkfu Chah

**Affiliations:** 1 Department of Veterinary Medicine, University of Nigeria Nsukka, Enugu State Nigeria; 2 Department of Animal Health, Federal College of Animal Health and Production Technology, National Veterinary Research Institute Vom, Plateau State, Nigeria; 3 Biotechnology Centre, National Veterinary Research Institute Vom, Plateau State Nigeria; 4 Transboundary Animal Disease Laboratory, Agricultural Research Council-Onderstepoort Veterinary Institute, Onderstepoort, South Africa; 5 Department of Veterinary Pathology and Microbiology, University of Nigeria Nsukka, Enugu State Nigeria

**Keywords:** colorimetric loop-mediated isothermal amplification, peste des petits ruminants virus, reverse transcription-polymerase chain reaction, small ruminant diagnostic tool

## Abstract

**Background and Aim:**

Peste des petits ruminants (PPR) is an acute, extremely contagious transboundary viral disease of small ruminants with severe economic consequences, caused by PPR virus. Cost-effective and rapid diagnosis of the disease is essential for prompt management and control. This study aimed to compare the application of a commercial colorimetric loop-mediated isothermal amplification (cLAMP) kit and reverse transcriptase-polymerase chain reaction (RT-PCR) in the diagnosis of PPR in sheep and goats in Southeast Nigeria.

**Materials and Methods:**

Nasal swab samples were collected from West African Dwarf sheep and goats showing clinical signs suggestive of PPR (n=80) and those without any clinical signs (n=140) of the disease. The diagnosis was achieved through detection of PPR viral genome in the samples using a cLAMP kit and RT-PCR. cLAMP assay was done directly on nasal swab samples without ribosomal nucleic acid extraction. A set of six primers targeting the matrix gene protein was used for the cLAMP assay.

**Results:**

PPR viral genome was detected by both cLAMP and RT-PCR in 51 (63.8%) of the 80 samples from sheep and goats with signs suggestive of PPR while 14 (10%) of those without signs tested positive for PPR by both assay methods. There was a 100% agreement in the cLAMP and RT-PCR results. However, cLAMP was a faster, easier, and less expensive method compared to RT-PCR.

**Conclusion:**

The cLAMP assay demonstrates the potential for a point of care diagnosis in the field and a valuable diagnostic tool in areas with poor electricity supply as well as in a less equipped diagnostic laboratory. Since the reagents are affordable, cLAMP can be a diagnostic tool of choice in the detection and surveillance of PPR virus in countries with limited resources.

## Introduction

Peste des petits ruminants (PPR) is a viral disease of small ruminants. It is acute, extremely contagious, trans-boundary, and a devastating disease that threatens livestock production in many developing countries [1]. It is caused by *Small* *ruminant* *morbillivirus* formerly known as PPR virus (PPRV) [2], a single-stranded ribosomal nucleic acid (RNA) virus which is a member of the *Morbillivirus* genus of the *Paramyxoviridae* family [1]. The disease is enzootic in several countries of West Africa, where it leads to extensive economic losses in small ruminant production [3]. The economic loss due to PPR outbreaks in Nigeria was estimated to be 42.5 million US dollar (USD) [4]. As a trans-boundary disease, PPR has been targeted for eradication by Food and Agriculture Organization (FAO) and World Organization for Animal Health by the year 2030 [1,5]. Presumptive diagnosis of PPR is usually based on clinical signs of fever, conjunctivitis, ocular discharges, encrustation in the median canthus, mucopurulent nasal discharges, ulcerative stomatitis, salivation, coughing, sneezing, profuse diarrhea, and dehydration with sunken eyes and emaciation [6]; thus, the disease can easily be confused with contagious caprine pleuropneumonia and other respiratory diseases which manifest same clinical signs. This makes the detection of PPR viral genome the standard for confirmation of the disease. Immunological and virus isolation are conventional diagnostic procedures for PPR. However, the slow, laborious, and insensitive nature of these conventional methods [7] necessitated the development of several nucleic acid-based techniques for PPR diagnosis. Among the several molecular techniques for the detection of PPRV, reverse transcriptase-polymerase chain reaction (RT-PCR) (the standard RT-PCR or real-time RT-PCR) is considered as a standard PPR diagnostic test globally [8,9]. However, this method requires expensive laboratory equipment (especially real-time RT-PCR) and skilled manpower and demands long working hours [10,11]. Consequently, the RT-PCR technique cannot be readily utilized in less equipped laboratories or in the field for routine diagnosis of PPR [12].

To overcome the problems and difficulties of PCR application in the field and less equipped laboratory, loop-mediated isothermal amplification (LAMP) was developed as a rapid one step, reliable, and sensitive single reaction temperature method for amplification of target gene sequence [13]. This technique has been employed for the detection of several pathogens such as parasites [14,15], bacteria [16,17], fungi and yeast [18], and viruses [19-24]. To facilitate visualization of the LAMP reaction, Tomita *et al*. [25] developed a simple colorimetric assay by addition of a fluorescent dye, calcein, to the pre-reaction solution. Simpler colorimetric assays for visual detection of the LAMP reaction by addition of colorimetric dye such as hydroxy naphthol blue [26] and malachite green [14] to pre-reaction solution have been reported.

In the previous application of LAMP protocol for PPR viral genome detection, purified RNA as a template was used, and addition of fluorescence dyes such as SYBR green and Eva green to the LAMP reaction mix enabled visual interpretation of results [19,24]. Thus, RNA extraction, purification, and conversion to cDNAs were undertaken before the LAMP. These processes increase the cost of diagnosis and lengthen the period of result turnover. LAMP diagnostic kits for several diseases including tuberculosis [27] and malaria [28] have been developed. WarmStart^®^ Colorimetric LAMP (cLAMP) 2× Master Mix Kit contains a colorimetric dye which enhances visualization of the LAMP reaction due to change of solution color [29].

This study was designed to compare the application of cLAMP kit and RT-PCR assay in the detection of PPR viral genome directly from clinical samples collected from suspected PPR infected and apparently healthy sheep and goats.

## Materials and Methods

### Ethical approval

This study was performed in line with the principles of the Declaration of Helsinki. Approval was granted by the University of Nigeria Nsukka Animal Care and Use Ethical Committee (UNN/eTC/16/82217).

### Sample collection

A total of 220 nasal swabs were collected from suspected PPR infected (n=80) and apparently healthy (n=140) West African Dwarf sheep and goats in Southeast Nigeria from December 2017 to June 2018. The animals had no history of PPR vaccination. The samples were transported in cold box to the Biotechnology Centre, National Veterinary Research Institute, Vom, Jos Plateau State, Nigeria, for analysis using cLAMP kit and RT-PCR.

### Sample processing and RNA extraction

Sterile phosphate-buffered saline (500 μL) was added into each tube containing the swab sample. This was centrifuged at 10,000× *g* for 3-5 min at 4°C. The supernatant (swab extract) was decanted into a sterile tube and stored at −70°C until needed for RNA extraction in the RT-PCR assay. Viral RNA was extracted from 140 μL of swab extract using the commercial viral RNA kit: QIAamp Viral RNA Mini kit (Qiagen, Hilden, Germany), according to the manufacturer’s instruction.

### The primer

The cLAMP primers comprised 2 outer, 2 inner, and 2 loop primers based on the matrix gene sequence of PPRV (Table-1), as described by Li *et al*. [19] while those for RT-PCR was based on N-gene sequence of PPRV (Table-2), as described by Couacy-Hymann *et al*. [8]. The primers were synthesized by Inqaba Biotech, South Africa.

**Table-1:** Primer sequences of matrix gene sequence of PPRV used for cLAMP as described by Li *et al.* [19].

Primer ID	Sequence description (5’-3’)	Position
Outer forward primer (F3)	TTGCAATGCAGTCAACCT	420-437
Outer backward primer (B3)	ATTCTCCCATGAGCCGA	620-636
Forward inner primer	GCACACTATAGTAACCATTGTCTGATGATACTCCCCAGA- GGTT	496-520
Backward inner primer	GGAGTTCCGCTCAGCCAATGTTCTAGGGTTTGTGCCATT	534-553
Loop forward	TCTAGTTATGCTCATGTACACAACC	468-492
Loop backward	GTAGCCTTCAACATCTTGGTTACAC	556-580

PPRV=Peste des petits ruminants virus, cLAMP=Colorimetric loop-mediated isothermal amplification

**Table-2:** Primer sequences of N-gene sequence of PPRV used for RT-PCR as described by Couacy-Hymann *et al.* [8].

Primer ID	Sequence description (5’-3’)	Position
NP3a Forward	TCT CGG AAA TCG CCT CAC AGA CTG	1232-1255
NP4 Reverse	CCT CCT CCT GGT CCT CCA GAA TCT	1585-1560

PPRV=Peste des petits ruminants virus, RT-PCR=Reverse transcriptase-polymerase chain reaction

### cLAMP KIT (WarmStart^®^ New England BioLabs Inc., UK)

cLAMP 2× Master Mix kit used contains a blend of Bst 2.0 WarmStart DNA Polymerase and WarmStart RTx RT in an optimized LAMP buffer solution. All components (WarmStart cLAMP 2× Master mix, Primer mix, dH_2_O and sample [nasal swab extract]) were thawed at room temperature. They were briefly vortex to mix and briefly centrifuged to remove drops of content from the inside of the lid and then placed on ice. The cLAMP reaction was carried out in a 25 μL volume mix (in accordance with the manufacturer’s instruction) containing 12.5 μL of WarmStart^®^ LAMP 2× Master Mix, 2.5 μL of 10× LAMP Primer Mix (contains 16 μM each of FIP and BIP, 2 μM each of B3 and F3, and 4 μM each of Loop F and Loop B), and 9 μL of dH_2_O and 1 μL of swab extract as template. For the negative control (without template), an equivalent volume of dH_2_O was added. This reaction mixture was dispensed into an Eppendorf tube, vortexed to mix and briefly centrifuged to remove drops of the mixture from the inside of the lid. The reaction solutions maintained a bright pink color due to the presence of phenol red dye. The Eppendorf tubes were then sealed and incubated at 65°C for 30 min using a heat block. The Eppendorf tubes were then removed from the incubation and examined by the naked eye for yellow coloration of sample in a positive test or pink coloration for a negative test [29]. The results were then photographed and recorded.

### RT-PCR

RT-PCR was carried out in 25 μL volume reaction mix using QIAGEN^®^ OneStep Ahead RT-PCR Kit (Qiagen, Hilden, Germany) consisting of 10 μL of 2.5× OneStep Ahead RT-PCR Master Mix, 1 μL of 25× OneStep Ahead RT-Mix, 1.5 μL of 10 pmol (each) of forward and reverse primers, 5 μL of Q solution, 3.8 μL of dH_2_O, 0.2 μL of 125× OneStep Ahead Master Mix Tracer, and 2 μL of template RNA. Aliquot of molecular grade water was used as the negative control while the positive control consisted of an established positive sample. Amplification was conducted under the following thermal conditions: 50°C for 10 min to activate the RT and 95°C for 5 min to activate the DNA polymerases followed by 40 cycles of 95°C for 10 s, 55°C for 10 s, 72°C for 10 s, and a final extension of 72°C for 2 min, according to the manufacturer’s instructions. The negative and positive controls were performed simultaneously with the test templates.

PCR amplified products (5 μL) were resolved on 1.5% agarose gel electrophoresis stained with ethidium bromide to reveal the expected band size. The gels were examined and photographed under ultraviolet light using a gel documentation system (BioRAd Gel DocTM xRT model no. Universal HoodII, BioRad, USA). The presence of a discrete band in the PCR product of the test sample that comigrated with the PCR product of the positive control indicated a positive result; while the absence of bands indicated the sample was negative. The size of the PCR products in the test samples and positive control was calculated (351 bp) by reference to the standard markers. The correct size estimation against a molecular weight marker was confirmed.

The rest 20 μL of the PCR amplified products from each sample that produced the expected band size following electrophoresis were submitted on dry ice to Trans-boundary Animal Disease Laboratory, Agricultural Research Council - Ondersteport Veterinary Research Institute, South Africa for purification, quantification, and subsequent submission to Inqaba Biotech South Africa for direct Sanger sequencing in both forward and reverse directions with primer pair NP3/NP4. Sequences were viewed using Chromas ver 2.6.4, then assembled and analyzed using BioEdit ver 7.2.5 software and submitted to the nBLAST program (https://blast.ncbi.nlm.nih.gov/Blast.cgi) to search and compare with other related sequences in public domain databases.

## Results

### Detection of PPR viral genome in nasal swabs of sheep and goat using cLAMP kit and RT-PCR technique

**Figure-1:**
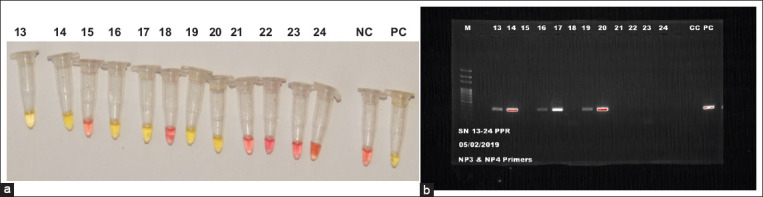
Results of representative samples using colorimetric loop-mediated isothermal amplification (cLAMP) and reverse transcriptase-polymerase chain reaction (RT-PCR). (a) Picture of color reaction of product produced by representative samples analyzed with cLAMP. Yellow color indicates positive reaction while pink color indicates negative reaction. CC and PC are the negative and positive controls, respectively. (b) Gel picture of amplification product produced by representative samples analyzed with RT-PCR. Lane M=100bp DNA molecular weight maker, Lane 13-24 are the test PCR product for representative samples. Lane CC and PC are negative and positive controls, respectively.

Out of the 220 nasal swab samples tested with the cLAMP kit, 65 (29.5%) showed color change from pink to yellow by visual examination of the endpoint cLAMP products using the naked eye (Figure-1a). Amplified products from same 65 samples that were positive in the cLAMP assay produced discrete bands on agarose gel that comigrated with the PCR product of the positive control (Figure-1b). Out of the 65 samples that tested positive for PPRV by RT-PCR, 38 good quality sequences were obtained by Sanger sequencing and they were comparable with PPRV sequences in the databases. These sequence data have been submitted to GenBank under the following accession number: MN271585 - MN271602 and MT038903 - MT038906. PPR viral genome was detected by both cLAMP and RT-PCR in 51 (63.8%) of the 80 samples from sheep and goats with signs suggestive of PPR while 14 (10%) of those without signs tested positive for PPR in both assay methods. Thus, there was a 100% agreement in the results of the cLAMP kit and RT-PCR technique.

### Comparative evaluation of cLAMP and RT-PCR methods of PPR detection

Based on duration and cost of analysis, RT-PCR consumed longer operational hours of about 4-5 h for endpoint gel electrophoresis while cLAMP required 30 min for endpoint visualization using the naked eye. RT-PCR requires purification of the viral RNA of the clinical samples and thus the use of sophisticated laboratory equipment (thermo-cycler, biosafety cabinet, etc.) while cLAMP was done directly on the clinical samples using water bath/heat block. The cost of processing one sample by RT-PCR (from RNA extraction to amplicon detection) and cLAMP (from swab extract to color change) was 6000 Nigerian Naira (NGN) (16.7 USD) and 1300 NGN (3.6 USD), respectively, (360 NGN=1 USD).

## Discussion

The 100% agreement in the results obtained with cLAMP kit and RT-PCR indicates that the cLAMP kit can be used as an alternative to RT-PCR as a primary diagnosis for PPR in an environment of limited resources. LAMP assay has been reported to be more sensitive than PCR [20]. The finding in this study agrees with the reports of previous authors [19,21,24] who employed the RT-LAMP technique to detect PPR viral genome in China, India, and Pakistan respectively. Furthermore, the LAMP assay was successfully used to detect herpesvirus of turkey from chicken samples in Nigeria [23]. The cLAMP kit also detected PPR viral genome in apparently healthy sheep and goats, indicating that the kit can be used to detect animals incubating the virus but without clinical signs.

The result turnover time for cLAMP was within 30 min revealing that the method is faster than the conventional RT-PCR which requires approximately 4-5 h for endpoint gel electrophoresis. This agrees with the report of other authors [19,23,24] indicating the robustness of the LAMP technique. It has been reported that LAMP amplifies the target viral genome within 1 h [22]. This, it does using isothermal nucleic acid amplification chemistry based on Bst polymerase strand displacement activity and use of a set of six primers (two outer, two inner, and two loop primers) [29].

In this study, PPR viral genome was successfully detected directly from clinical samples without RNA purification. This is in line with the report of Ashraf *et al*. [24] who detected PPR viral genome using RT-LAMP. However, RT-LAMP assay reported by the previous authors [19,21,23] used purified RNA/DNA. Isolation of purified RNA/DNA essentially demands a sterile environment and sophisticated laboratory equipment and conditions (like an expensive and lengthy process of RNA extraction), and this limits the application of RT-PCR and RT-LAMP in the field and poorly equipped laboratories in resource-poor country.

The troubles/problems of optimization of the RT-LAMP reagent are not a feature in cLAMP [29], as the cLAMP mix is an optimized formulation of Bst 2.0 WarmStart DNA polymerase and WarmStart RTx in a special low buffer reaction. The previous reports on RT-LAMP required an addition of a fluorescent dye like SYBR green or Eva green into the LAMP reaction [17,19,24] for end-product evaluation with the naked eyes or the use of 2% agarose gel electrophoresis stained with ethidium bromide [19,23,24] where ladder-like bands confirm positive samples. These expensive dyes consequently increase the cost of the RT-LAMP assay, while the electrophoresis procedure and gel imaging lengthen the duration of the RT-LAMP procedures. cLAMP reagent/mix contains a pH indicator such as phenol red for rapid and easy detection of target genome and interpretation of results, as a change of color from pink to yellow indicates a positive sample [29]. cLAMP is a single tube reaction assay that does not require opening of the reaction tube before reading the results; thus, post-amplification contamination leading to false positive results is avoided.

The cLAMP assay implemented in this study was robust as the technique provides a straightforward and rapid way of detection of PPR virus genome in clinical samples. Speedy diagnosis with simplicity remains a crucial factor in the diagnosis of a disease condition in the field and also can be extremely efficient when applied in disease control strategies which can lead to the eradication of the disease. PPR has been targeted for eradication by 2030 by the WHO and FAO [5], and this can only be achieved by effective enforcement of the disease control strategies. These control strategies require monitoring of the disease circulating in the concerned population through timely, sensitive, and efficient diagnostic methods [30]. In resource-poor countries with epileptic power supply, there is a need for this technique (cLAMP) to be explored. This will indeed improve the quality of veterinary service delivery and consequently, control the spread of diseases through timely intervention. It is worthy to note here that the major limitation of the cLAMP technique is the storage of the LAMP Master Mix which requires −20°C; which can be a challenge in an area with epileptic power supply. However, such limitation can be overcome by the acquisition of solar operated freezers.

## Conclusion

The cLAMP kit used in this study has proven to be a reliable alternative to RT-PCR in the rapid detection of PPR infection in sheep and goats. It has achieved one of the goals in veterinary diagnosis: Diagnosis should be affordable, sensitive, specific, user-friendly, robust, equipment-free, and deliverable to the end-user. The cLAMP assay is helpful to those working in areas of limited resources in terms of electricity, laboratory equipment, and funds for reagents. This is the first report of the use of cLAMP for the detection of a viral genome of veterinary importance directly from clinical samples in Nigeria.

## Authors’ Contributions

ICC, EIU, and KFC contributed to the study conception and design. Material preparation, data collection, and analysis were performed by ICC, KIO, PDL, RPM, and LEH. The first draft of the manuscript was written by ICC, which was revised and edited by KFC and ICC. All authors read and approved the final manuscript.
